# A transdisciplinary approach to the decision-making process in extreme prematurity

**DOI:** 10.1186/1756-0500-7-450

**Published:** 2014-07-14

**Authors:** Marc Simard, Anne-Marie Gagné, Raymond D Lambert, Yves Tremblay

**Affiliations:** 1 Cellular and Physiological Sciences, Faculty of Medicine, University of British Columbia; Life Sciences Institute, Rm 2.320, 2350 Health Sciences Mall, Vancouver, BC V6T 1Z3, Canada; 2 Obstetrics, Gynecology & Reproduction, Faculty of Medicine, and Centre de Recherche en Biologie de la Reproduction, Laval University; Centre Hospitalier Universitaire de Québec (CHUQ) Research Center, Rm T-1-49, 2705 Laurier Blvd, Quebec City, QC G1V 4G2, Canada

**Keywords:** Transdisciplinarity, Web-based forum, Neonatology, Prematurity, Sex differences

## Abstract

**Background:**

A wide range of dilemmas encountered in the health domain can be addressed more efficiently by a transdisciplinary approach. The complex context of extreme prematurity, which is raising important challenges for caregivers and parents, warrants such an approach.

**Methods:**

In the present work, experts from various disciplinary fields, namely biomedical, epidemiology, psychology, ethics, and law, were enrolled to participate in a reflection. Gathering a group of experts could be very demanding, both in terms of time and resources, so we created a web-based discussion forum to facilitate the exchanges. The participants were mandated to solve two questions: “Which parameters should be considered before delivering survival care to a premature baby born at the threshold of viability?” and “Would it be acceptable to give different information to parents according to the sex of the baby considering that outcome differences exist between sexes?”

**Results:**

The discussion forum was performed over a period of nine months and went through three phases: unidisciplinary, interdisciplinary and transdisciplinary, which required extensive discussions and the preparation of several written reports. Those steps were successfully achieved and the participants finally developed a consensual point of view regarding the initial questions. This discussion board also led to a concrete knowledge product, the publication of the popularized results as an electronic book.

**Conclusions:**

We propose, with our transdisciplinary analysis, a relevant and innovative complement to existing guidelines regarding the decision-making process for premature infants born at the threshold of viability, with an emphasis on the respective responsabilities of the caregivers and the parents.

## Background

A premature delivery is a very stressful and emotional situation for the parents. In this context, several questions on the survival of the baby and the potential consequences of a premature birth arise. Parents could ask themselves if they share the same opinion on the situation and if it is possible for them to make a clear decision in such hard times. It might also be difficult for the health care providers to have a global view of such a sensitive situation, when the future of the patient remains so uncertain. Issues related to several fields of knowledge have to be considered, including clinical, ethical and sometimes legal concerns thus making the knowledge of a single professional limited.

In recent years, translational studies promoting a transdisciplinary approach became more widely used in health research. Transdisciplinary research and education require a strong collaboration between open-minded members of various disciplinary fields to resolve complex issues, such as those encountered in the context of prematurity. Once the disciplinary barriers have been crossed, a holistic understanding of the issue can be achieved [1]. This transcends the first level of comprehension of biological and medical content and is more likely to meet the societal values and needs.

Gathering a group of experts from various sectors (academic, private, and/or public) with the aim of solving a problematic using a transdisciplinary approach could be very demanding, both in terms of time and resources [2]. Indeed, reaching a unified vision on an issue may require long and frequent discussions. Also, the more complex the problem, the more aspects it involves, and therefore the greater the number of disciplines needed to resolve it. Finally, most experts have busy schedules and can’t afford frequent meetings, especially if they have to travel to meet each other. All those factors could delay the process or lead to the abandonment of the project. However, several technologies allow the participants to overcome those obstacles. For example, teleconference, email, web-based videoconference or web-based forum can be used to join people wherever they are. A virtual meeting space as a mean to cross barriers in a transdisciplinary study has been tested previously and has led to conclusive results [3]. In that web-based discussion forum, participants succeeded in unifying their vision on a very prickly question regarding the frequency of multiple births resulting from the transfer of multiple in vitro fertilized embryos.

A second web-based transdisciplinary forum was launched to address questions in the context of extreme prematurity, to take advantage of and further validate the usefulness of this kind of tool to resolve complex issues. Although recommendations for the management of extreme prematurity have been published, we present here novel elements that emerged from our transdisciplinary approach, especially regarding the choice and responsibility of the parents, the criteria to use to decide to reanimate or not an extremely premature infant, the consideration of the infant’s sex as a risk factor, and the involvement of a multidisciplinary team in the decision process.

Experts from several fields, namely epidemiology, biomedical sciences, psychology, ethics, and law, were involved in the process. They had to answer these questions: What are the parameters to consider before delivering survival care to a baby born at the threshold of viability? Would it be acceptable to give different information to parents according to the sex of the baby considering that outcome differences exist between sexes? First, the present experiment shows how a web-based transdisciplinary forum and its advantages (e.g. no physical meeting, time flexibility, etc.) can be a useful tool in knowledge translation. Second, we present the transdisciplinary report generated through this methodology, that includes updated biomedical and epidemiological data about extreme prematurity, the respective responsabilities of parents and caregivers, ethical principles with an emphasis on the best interest of the child, legal aspects, as well as several psycho-social elements.

## Methods

### Discussion forum format

People from different geographical locations can interact through videoconference or chatting room, but timing constraints may be problematic. In contrast, a web-based discussion forum presents many advantages. Everyone can read from other disciplines or transmit his own knowledge whenever he wants to, thus eliminating constraints related to atypical/variable/loaded schedule, time zone, and location. It also allows the recruitment of people from all over the world, which helps to find qualified experts. Importantly, the fact that participants can read threads unlimitedly is helpful to get a deeper knowledge and comprehension of others’ thoughts.

Forum administrators had to build the forum’s guidelines, to stimulate the exchanges and to ensure that the debate was staying on the right track. The forum was built on a WebCT platform and hosted by Laval University-Québec City. Every participant was given an identification code to access the discussion board. All guidelines were deposited by the forum administrators under a “didactic material” section. Then, participants were allowed to post and create new threads. Every new document relevant to the discussion was also posted on the forum.

This project was funded by a grant (#GTA92185) from the Canadian Institutes of Health Research (CIHR) and while other specific projects included in this grant application obtained the approval of an ethics committee (CHUL Research Center Research Ethics Committee; project #126.05.03), the web-based discussion forum as a tool for formation and knowledge translation did not require a specific approbation by the ethics committee.

### Recruitment

#### Forum participants

Among the advantages of a web-based forum, the absence of physical meeting and the time flexibility both considerably facilitated the recruitment of experts that were contacted by phone and email. Given the nature of the problem, experts from the following fields were invited to participate to the discussions: 1) epidemiology, 2) biomedical, 3) psychology, 4) ethics, and 5) law. To offset the lack of an expert from the medical field, a meeting with neonatologists and a visit at the neonatal intensive care unit (NICU) of the Laval University Hospital Center (CHUL) were scheduled (see details below). The composition of the work team was: one student in epidemiology (Québec City, CAN), six students (Québec City, CAN) and two post-doctoral fellows in biomedical sciences (Québec City, CAN and Rouen, FR), one student in experimental psychiatry (Québec City, CAN), and one student in bioethics (Rimouski, CAN). Two professional lawyers also participated (Montpellier, FR and Sherbrooke, CAN). Student members of the biomedical team were all performing graduate studies under the direction of one of the forum administrator, and their participation was a part of their curriculum. Participants gave a verbal consent to participate to the forum, and some of them were enrolled in a graduate course at Laval University (ETH65453) on transdisciplinary training.

#### Mentors

University teachers/researchers working in the following disciplinary fields were invited to participate: biomedical, ethics, psychology, and epidemiology. Their role was to ensure that the information shared on the forum was accurate and as exhaustive as possible. They were also instructed by the forum administrators to intervene minimally to avoid influencing the course of the discussions. They were more closely involved during the redaction of the unidisciplinary reports, and also had to approve the transdisciplinary statement.

### Timetable and evaluation

One of the most important advantages of the forum was the schedule flexibility, but the total duration of the forum exchanges had to be pre-determined to produce results in a timely manner. Therefore, the forum lasted for a total of nine months: from September 2008 to June 2009. It was divided into three phases (uni-, multi/inter-, and transdisciplinary). The first two months were devoted to the unidisciplinary exchanges. During the first two weeks, participants had to introduce themselves and to answer spontaneously to the questions asked in the forum (see Background), based on their professional experience and personal feelings. During the following month, all participants were required to familiarize themselves with the literature that was relevant, from their disciplinary perspective, to the questions asked in the forum and to prepare a summary document. At this step, each discipline was expected to work on its own to address the questions [4]. At the end of the first phase, five documents reviewing the epidemiological, biomedical, psychosocial, ethical, and legal aspects of the questions were produced. Once the mentors approved these documents, they were posted on the forum. This was the beginning of the multi- and interdisciplinary phase that was intended to last six months. Each document had to be read by all participants, and then had to be collectively discussed and criticized. Participants had to address their concerns about data and points of view presented by the other disciplines. During the discussion phase of the forum, each participant had to contribute 1–2 times a week and to keep track of the ongoing discussions. This step was crucial to understand and assimilate the disciplinary perspectives of the issues and to develop a more comprehensive vision. During this phase, participants should translate disciplinary knowledge into information that is accessible to other participants, establish a frank dialogue (the capacity to frankly answer questions raised by other participants and propose solutions), develop curiosity for the perspectives and concerns of others, cross disciplinary barriers (to understand and use the language of others), and to detach themselves from their initial opinion to build up a final opinion that reflects the concerns of all disciplines. If performed adequately, this phase should lead to the transdisciplinary level, to the integration of the knowledge [5]. Even though it may be difficult to clearly distinguish the transition between the last two phases, the transdisciplinary phase was intended to last one month. The forum was closed with the redaction of a transdisciplinary statement presenting the pro and con arguments related to the questions of the forum, based on the analysis and integration of the knowledge and perspectives from all the disciplines involved. Participants and mentors had to review the transdisciplinary report to ensure that a consensual opinion was reached. For instance, the participants were asked: Do you agree with the final report? Is the content in accordance with the information exchanged in the discussion forum? Do you feel that your opinion was taken into consideration in the final report? Altogether, we estimate that the total time spent by each participant was approximately 60 hours, although this was variable because some unidisciplinary documents took more time to write than others and four participants had to draft the transdisciplinary report.

## Results

### Phases

#### Unidisciplinary phase

All participants met the schedule; disciplinary documents (10–15 pages) were prepared following an exhaustive review of the relevant literature. The mentors approved the disciplinary reports of their respective field. A summary of the observations made by each disciplinary field is presented in Table 1. For further information on disciplinary reports, please see [6,7].

**Table 1:** Summary of the main observations on prematurity and the influence of fetal sex from unidisciplinary fields

**Field**	**Main observations**
Epidemiology	-The major causes of prematurity are: planned premature delivery for maternal or foetal reasons, spontaneous preterm labor, and preterm premature rupture of membranes.
	-A birth at 22–24 weeks is considered at the limit of viability, with 11-30% survival at 23 weeks, 54-76% at 25 weeks, and 90-95% at 28–31 weeks.
	-Risk factors of prematurity related to the mother: maternal age, infections, in vitro fertilization, background of premature delivery, smoking, psychosocial background.
	-Risk factors related to the foetus: intra-uterine growth restriction, multiple births, males are at higher risk of premature birth.
	-The use of antenatal corticosteroids reduces the mortality rate and the risk and severity of respiratory distress syndrome, cerebral hemorrhage, and necrotizing enterocolitis.
Biomedical	-Main organs at risk in prematurity: skin (homeostasis), eyes (retinopathy), cardiovascular system (arteriovenous shunt), intestine (necrotizing enterocolitis), brain (cerebral palsy, hemorrhage), lung (respiratory distress syndrome, bronchopulmonary dysplasia).
	-Respiratory distress syndrome is a main concern, with males being more susceptible than females due to a delay in lung maturation that is related to androgens.
	-Some lung maturation biomarkers can be tested in the amniotic fluid, but this is not always feasible and/or useful in the clinic.
	-Commonly used treatments: antenatal corticosteroids have well documented positive effects on the maturation of the surfactant system; the use of surfactant mixtures and assisted ventilation protocols greatly improves outcomes.
Psychosocial	-Cognitive sequelae of prematurity: some retardation at preschool (2–3 years) and school age (6–8 years), with a male disadvantage. Not much difference is observed in school performance at 10 years old. In adolescents and young adults, some high-level cognitive functions (suppression of automatic responses, mental flexibility) may be affected and translate into learning problems in school.
	-Behavioral and affective sequelae of prematurity: deficits are more prevalent at preschool age, and psychiatric disorders (especially attention deficit hyperactivity disorder) are more prevalent at school age, with sex differences in type and severity. In young adults, some confidence and behavior problems are reported, with a male disadvantage..
	-At cognitive, behavioral, and affective levels, most “problems” tend to decrease with age.
	-In parents, prematurity can lead to high stress, anxiety, and depression, especially when the child has severe health problems. The stress experienced by parents can negatively impact on the development of the child.
Ethics	-Technical advances lead in some cases to extended time to death; use of invasive treatments that may or may not be beneficial.
	-Importantly, an initial reanimation/life-support, that can be temporary, will allow time to evaluate the situation.
	-Main types of prioritization of risks: above all, avoid wrongfully stopping treatment; avoid wrongfully continuing treatment; above all, avoid damage.
	-The risk of heroic measures is always present and has to be considered.
	-Main ethic principles involved: beneficience, autonomy, best interest of the child (to be considered by parents and physicians), free and enlightened consent, respect of human life and quality of life, justice and equity, precautionary principle.
	-The communication between parents and physicians and the involvement of parents in the decision process are of high importance.
	-Palliative/confort care should be provided if needed.
	-Unfortunately, decisions made in the so-called “grey zone” can be mistakes.
Law	-Legal status of the premature infant: the legal status begins and ends with life, and associated civil rights are the right to life, to inviolability, and to bodily integrity. According to Canadian Law, the foetus does not have a legal status. The child becomes a person when out of the mother’s body, alive, and viable; breathing or not, with independent circulation or not, umbilical cord severed or not. A medical evaluation is required to assess the viability of the child. The legal status is a prerequisite to be eligible to receive treatments.
	-Rights and duties of the parents: parental authority, free and enlightened consent to treatments or arrest of treatments, in the best interest of the child.
	-Duties and obligations of the physicians: duty to inform, obligation to treat when consent is obtained.
	-It is possible to contest a decision (parents against physicians, or vice versa) in a court of law if needed, where the judge will render a judgement in the best interest of the child.
	-Some shifts exist between law and scientific knowledge.

#### Multi- and interdisciplinary phase

This phase was also successfully completed. Nearly 200 exchanges have been posted on the website. This reflected a great interest for the subjects under discussion; also, participants were curious to know beyond the information provided by the unidisciplinary documents. The respectful and enthusiastic attitude toward the questions and comments demonstrated the open-mindedness of the participants. They were not arguing in a negative manner against each others and were curious about the perspectives of other disciplines. Mentors only had to intervene sporadically during the discussions, showing that the participants were doing excellent without external help and demonstrating the maturity of the group. The only issues encountered during this phase were: 1) the discussions sometimes diverted from the original topic, creating a waste of time, although the participants were easily put back in the right direction by the forum administrators, and 2) some participants tended to contribute more than others.

#### Transdisciplinary phase

The participants took about one month to integrate the multidisciplinary knowledge. According to the criteria mentioned in the Methods, the nature of the participants’ comments led us to believe that a transdisciplinary level was achieved. For instance, many of them referred to the knowledge of a colleague or to a conclusion that was collectively made during the interdisciplinary phase. Also, when participants had to negotiate, the process went in a respectful way. Further, those examples correspond to the principles 3 and 4 of the Klein’s framework of transdisciplinarity evaluation (i.e. “leveraging of integration” and “social and cognitive factors interaction”) [8]. Once the participants thought that a transdisciplinary state of understanding was achieved, a review paper was written. Three members of the biomedical team and the epidemiological expert proposed themselves to do this, as it was a more convenient and faster way to proceed. Over a period of one month, three versions of the report have been written and reviewed by the mentors and other participants. The aforementioned criteria were also met adequately in the written report.

### Overpassing the initial objectives

Even though the objectives of the forum were met, the participants decided to go further and to publish their work. After having been adapted and reviewed to make them more understandable for a larger audience, including parents of premature children, unidisciplinary documents and the first version of the transdisciplinary statement were published as an electronic book (eBook) in both French and English [6,7]. This involved not only the forumers and the mentors, but also a scientific reporter (Mr. Jean-Pierre Rogel, Radio-Canada).

### Participant satisfaction

A main goal of the discussion forum was to validate the capacity of such a procedure to solve multifaceted problems. However, a high level of satisfaction of forumers is crucial to keep the discussion ongoing. We therefore thought that it was important to evaluate this parameter at the end of the activities. No formal scale (e.g. Likert-type, a scale on which the participant rates his degree of agreement with a statement [9,10]) was used. Instead, participants were asked to report whatever they thought about the forum. Then, the individual semantic content was categorized as “positive”, “negative” or “neutral”. For example, in “I was disappointed I could not contact a colleague in private”, the word disappointed was categorized as negative, while “I learned a lot” was categorized as positive. Overall, the positive content far exceeded the negative one. Recurrent comments were made about the open-minded nature of the experiment such as: “we learned that no discipline is more important than another”, “it improved our team working skills”, “we are now able to criticize our own disciplinary field”, “we are now more curious about the knowledge of others”, “we are now more attentive to the need to communicate and transfer scientific knowledge”. Other comments were more negative. For example, one of the major complaints was about the impossibility of contacting other forumers in private. This might have bothered certain participants when some debates were related to the forum but not necessarily appropriate. An example of this was a debate about late-abortion.

## Discussion

### The transdisciplinary approach

Regarding the unidisciplinary phase, all the participants met the initial goal (redaction of individual reports approved by the mentors) so we consider that this first step was successfully completed.

The participants went through the multidisciplinary phase quite well, but the forum did encounter some issues. First, many participants and especially those from the biomedical team raised a concern about the popularization of the scientific language. Indeed, experts from all disciplines had to make some efforts to communicate the information they wanted in a lay language without distorting its meaning and accuracy. The second difficulty encountered in this phase was the absence of clinician among the participants, making the process to slow down at some point. As the number of questions requiring a clinical opinion was growing, participants knew they were not going to solve some issues without the input of professionals who were dealing with preterm infants and their parents on a daily basis. To overcome this situation, a meeting was organized during which two neonatologists answered a list of questions created from a review of the posts of the forum. In addition, the neonatologists suggested that it would be worthwhile for the participants to visit the NICU to “get in touch” with their debate. The participants really appreciated the visit, were globally satisfied of the answers, and were then ready to pursue the discussions. One last difficulty in this phase was that some participants contributed more than others. This discrepancy between disciplinary inputs during multidisciplinary exchanges may have had an impact on the final report, although potential biases have been minimized during its redaction (see below).

As indicated above, three members of the biomedical team and the epidemiologist wrote a transdisciplinary report. Three preliminary versions have been successively reviewed by the mentors as well as other forum participants and most of the changes were semantic-related or related to aspects that were not addressed during the multidisciplinary phase. It was then considered that the authors of the transdisciplinary statement did reached the transdisciplinary goal. All forumers were pleased by the final result and confirmed that it represented their own new vision, thus indicating that an unified opinion was reached.

The present experiment confirms that it is possible for a group of experts from different disciplinary fields to get a unified vision of a multifaceted and complex issue, in a timely manner. It also demonstrates that it is feasible to do so without constraining schedule and with minimal need to travel. In addition, an electronic book has been published, thus showing that the knowledge produced through this forum got accessible to a larger audience than it was initially thought.

Even if we consider this experiment as globally successful, some parameters will need improvement. First, due to the absence of a clinical expert on the discussion forum, it was not possible to reach our goal without a physical meeting between participants and perinatal practitioners. Therefore, a future web-based translational forum would benefit from spending more effort on the recruitment of participants, to ensure that all disciplines required for resolving specific issues are present at the beginning of the exchanges. In this way, a transdisciplinary approach of a problematic may be achieved in a complete virtual environment. Second, most of the forumers have studied in fields of knowledge more or less related to prematurity. Would the experiment have turned out differently if someone representing the general population, devoid of any kind of disciplinary or scientific paradigms, had participated to the forum? Also, would we have benefited from the opinion of parents of preterm infants?

It has been possible in the present study to identify the transition from unidisciplinary to transdisciplinary understanding of the questions. Indeed, following the translation of knowledge through frank and interested discussions, the disciplinary barriers have been crossed. The disciplinary fields questioned each others until a consensual opinion was reached. Further, the final statement is accessible to anyone interested in the subject. However, the quantification of the degree of evolution toward transdisciplinarity throughout the forum could be improved. To do so, a future forum should use quantifying instruments, as several have been developed over the past years [11-13].

### The transdisciplinary analysis

There is no formal procedure or code in neonatology units in respect to the decision process regarding an infant born at the threshold of viability (the range of gestational age at birth, typically 23–24 weeks of gestation, where the risks of mortality and potential adverse consequences are very high), although several papers have proposed guidelines for the management of this delicate situation [14-17]. The application of these recommendations may vary between medical centers and probably from physician to physician. Guidelines and legal frameworks also differ from a country to another [18,19]. Notably, the level of participation of the parents in the decision process, as well as its perceived importance, seem to vary greatly. We present here an improved and updated version of the innovative analysis that resulted from our transdisciplinary approach on the subject. A summary of the analysis, and therefore the answers to the questions asked in the forum, are presented in the list below.

#### Highlights of the transdisciplinary analysis

● It should be recognized that to date, no strict recommendations exist for the management of a birth at the threshold of viability; here, we aim to contribute to the reflection, add elements for discussion, and complement available guidelines on the topic.

● An initial life-support/reanimation of an infant as a mean to allow time for evaluation, reflection and discussion, should be considered.

● The parents have to be closely involved in the decision-making process, as part of their legal and ethical rights and duties.

● The best interest of the child should be central for both parents and physicians.

● The fetal sex should be considered, among several factors, in the decision process in relation to poorer outcomes for males. It is ethically and legally acceptable to do so and it contributes to a free and informed consent.

● The involvement of a team of caregivers (obstetrician, neonatologist, neonatal nurse, psychologist, social worker) should be considered in discussions with parents. Further, there is a need for consensus within the team of caregivers.

The medical team has the responsibility to inform the parents on the health status of their premature infant and on potential associated consequences, which is a difficult and often underachieved task [20,21]. In the moments before and after birth, the data regarding the health status of the infant are often limited. The medical team must rely on available clinical and epidemiological data to inform the parents. These include information on the mother (e.g. age, hypertensive disorders, background of premature delivery) and on the infant (e.g. gestational age, weight, sex). Pharmacological interventions on the mother and/or the baby are also considered. Of note, an algorithm designed to predict outcomes, that considers gestational age, birth weight, gender, use of prenatal steroids, and singleton pregnancy, may be useful to the caregivers [22]. In addition, the parents and the medical team have to consider the accessibility to specialized healthcare in neonatology and the experience level of the caregiving team, that may be different from a hospital to another.

The reanimation of an extremely premature infant may lead to several short-term adverse consequences, for instance respiratory distress syndrome and intraventricular haemorrhage. Potential long-term consequences include chronic respiratory problems and cerebral palsy. In addition, extremely premature infants are more prone to suffer from mental and developmental retardation, delayed communication and competence acquisition, in addition to attention deficit, lack of self-confidence and affective issues [6]. At comparable early gestational age, the premature boys are at greater risk than girls to develop these consequences. However, it is generally admitted that the latter fact also apply for the “born full-term” population. Finally, even in the absence of any noticeable physical or mental sequel related to prematurity, premature infants are more at risk to be bullied at school [23].

It may be very difficult for parents to take an informed decision because, even if they are judges of the situation, they are directly involved in it. The decision to be made will not only have an impact on the life of the infant, but on theirs as well. This situation may lead to anxiety, depression, exhaustion, and problems in the couple. According to the law, at least in Canada, the parents are the only persons responsible for decisions regarding reanimation, treatments, or withhold of treatments. The parental decisions must be taken in the best interest of the infant. In theory, the medical team must limit itself to its ethical and legal duty that is to inform the parents on the health state of the child, on potential consequences on its development, on possible treatments, and on palliative (comfort) care if needed. Also, to share the uncertainty of the prognosis with the parents appears important. To facilitate the transmission of information, a prenatal consultation form template and a decision-aid in the format of a set of cards that contains pictographs and short texts are available and may be useful [14,24]. It is noteworthy that the opinions and attitudes of the different caregivers (neonatologists, obstetricians, residents, neonatal nurses) toward resuscitation and outcomes were shown to differ within them as well as between them [17,25,26]. This highlights the need for consensus within the medical team to avoid misunderstanding and confusion in both staff and parents. In addition, the medical team has to be careful in the way information is provided because message framing (positive vs negative) was shown to influence decisions [27] and also because the information, and its relative importance, may be interpreted differently between caregivers and parents [28-31]. The personal values of the parents have to be considered in the discussions, but nevertheless the process should lead to an informed decision that has to go beyond, for example, hope or spiritual beliefs. In some situations, the involvement of social workers and psychologists in the decision process, along with the caregiving team and the parents, may be relevant. In the process, caregivers also have the difficult task to assess the validity of an informed consent from the parents. If the medical team believes that the parents are not taking a decision in the best interest of the child, it can refer the case to a court of law that will render a final decision.

In some circumstances, the primary reanimation of an infant, in order to give time to the caregivers to define the prognosis and let the parents make their decision, should be considered. This situation may arise when clinical data are contradictory, incomplete, or when parents are not decided or disagree on the decision to make. The decision can therefore be made later when more information is available and after having had some time for reflection and discussion, without the initial pressure. A primary reanimation, that can be temporary, facilitates a free and informed decision-making from the parents and this approach seems highly relevant to us.

The prognosis for a premature infant has many limits. Actual epidemiological and clinical parameters cannot predict with certainty the presence or severity of outcomes for one given premature baby. For instance, there is no criterion to accurately predict before birth the severity of an eventual respiratory distress syndrome, even if several risk factors have been identified. Even though premature boys have a poorer prognosis than girls, it does not mean that every boy will have bad outcomes. Also, the efficacy of a treatment or the severity of the adverse effects linked to a given treatment cannot, at the individual level, be predicted with certainty. For instance, antenatal glucocorticoid treatments show variable efficiency, with some infants that even show no response to the treatment [32], and may cause neurological problems of variable intensity. New parameters and prognosis tools are under development to define more accurately the physiological status of a premature infant. Interestingly, a standardized template for clinical studies on preterm birth has been recently proposed and the adoption of such methodologies by the research community would facilitate the generation of meaningful meta-analyses [33]. A more personalized approach according to the specific condition of the infant, to minimize the consequences of prematurity, is wished. Indeed, it is crucial to stress out the necessity to develop better prognosis tools that will also facilitate the decision process.

The responsibility of the caregiving team is not limited to treat the infants and to inform the parents. The objective is not solely to save the infant’s life, but to take all means necessary to minimize the negative consequences and promote an acceptable quality of life. Therefore, regarding premature infants born at the threshold of viability, the medical team faces principally two objectives, namely the protection of life and the maximization of quality of life. The medical team has the responsibility to apply these two principles in the best interest of the child. In the recommendations given to parents about the continuation or the interruption of treatments, the physician bases himself on ethical principles to rationally analyse all factors involved in the decisional process. These principles include the respect of human life, the respect of a person’s autonomy, and the principle of benevolence and non-malevolence (the so-called “treatment dilemma”).

In our transdisciplinary forum, we also asked if it would be acceptable to provide information to parents according to the sex of the baby. As mentioned, some consequences of an extremely premature birth may be worst for boys, although these cannot be predicted with certainty. Further, it is not clear how clinicians present this risk factor and how parents perceive it and include it in their reflection before giving an informed consent on treatments. Also, it may be delicate to pose a different prognosis according to the sex of the baby considering that equity between men and women is a great concern in our modern society. However, in the context of extreme prematurity, it is ethically and legally acceptable to give parents medical information in function of the infant’s sex. Indeed, to give incomplete information is against a free and informed consent and against the medical code of deontology. Conversely, a decision solely based on the sex of the infant would be inappropriate. Here, one must not perceive sex discernment as a negative discrimination, but rather as a possibility to offer a more adapted intervention to each patient.

## Conclusions

The results of the present experiment confirm that a web-based discussion board can be a convenient, low cost, and effective way to help specialists discuss multifaceted issues. In addition, a benefit was reported about improvement of team working skills that can be very useful in any domain.

With the growing efficacy of new health treatments, saving life in unusual or infrequent conditions will become easier. Therefore, it is reasonable to think that more questions about what should be considered an "acceptable threshold" regarding life or quality of life will continue to rise. The present forum focused on prematurity and its consequences, but a lot of other complex questions on topics such as assisted suicide, palliative care, genetic counselling, personalized medicine as well as other non-health related questions would require experts to work together to improve decision-making processes.

## Competing interests

The authors declare that they have no competing interests.

## Authors’ contributions

MS and AMG participated in the analysis and wrote the manuscript. RDL and YT participated in the design and coordination of the study and helped to draft the manuscript. All authors read and approved the final manuscript.

## Authors’ information

Dr. MS is interested in steroid hormone metabolism and transport, especially during fetal development and in relation to prematurity. Dr. AMG is working in psychiatry genetics and ophthalmological research. Dr. RDL is an emeritus researcher of the Centre de Recherche en Biologie de la Reproduction, Laval University. His research focused on foeto-maternal interactions and he was interested by bioethics, especially in regards to in vitro fertilization. Dr. YT is the director of the Fonds de Recherche du Québec en Santé (FRQS)-Respiratory Health Network. His research focuses on steroid hormone metabolism and sex differences in the context of prematurity and related respiratory diseases.
